# Effect of Nutritional Guidance on Oral Function in Patients Visiting a General Dental Clinic: A Preliminary Study

**DOI:** 10.3390/jcm15010023

**Published:** 2025-12-19

**Authors:** Kazuki Makita, Takahiro Ono, Akiyo Kawamoto, Kazuya Takahashi

**Affiliations:** Department of Geriatric Dentistry, Osaka Dental University, Osaka 540-0008, Japan; makita-k@cc.osaka-dent.ac.jp (K.M.); kazuya-t@cc.osaka-dent.ac.jp (K.T.)

**Keywords:** nutrition, oral, function, body composition, dental clinics, mastication

## Abstract

**Objectives**: This non-randomized and self-selected controlled intervention study aimed to examine the effects of simple nutritional guidance provided by registered dietitians on the oral function and body composition of patients in a general dental clinic in Japan. **Methods**: Among patients aged ≥50 years who visited a single dental clinic for regular maintenance, those who accepted and declined nutritional guidance were classified as being in the “guidance group (G group)” and “nonguidance group (nG group)”, respectively. The oral moisture content, masticatory performance, tongue/lip motor function, maximum tongue pressure, and swallowing function were assessed. Additionally, we assessed 10 body composition parameters using a body composition analyzer. Patients in the G group received monthly nutritional guidance from a registered dietician. At 3 months, changes in oral function and body composition were examined and between-group comparison in changes in dietary habits was performed. **Results**: A two-way analysis of variance revealed a main effect of time and an interaction between time and group for oral function only, and improvements in masticatory performance scores, maximum tongue pressure, and swallowing function were observed in the G group (n = 20). Compared with the nG group (n = 18), the G group had a significantly higher proportion of participants with a reported increase in the variety and amount of consumed food. **Conclusions**: Although this is the preliminary trial with a small sample and high risk biases, our findings suggest the possibility that 3 months of nutritional guidance at a general dental clinic in Japan may improve oral function.

## 1. Introduction

With the rapid aging of the Japanese population, there has been increasing attention on the impact of declining oral function on overall health and quality of life [1,2,3,4,5]. Accordingly, early interventions for maintaining and improving oral function from middle age onwards have become increasingly important. Against this backdrop, the disease concept of “oral hypofunction syndrome” [6] was included in health insurance coverage in 2018, with the target age group being reduced from ≥65 years to ≥50 years in 2022 [7,8].

In order to maximize the effectiveness of these policies, it is necessary to systematically and interactively analyze how oral function is related to nutritional intake, lifestyle-related diseases, and frailty, and to develop possible countermeasures. There is a reciprocal relationship between oral function and nutritional status [9]. Several factors contribute to reduced chewing ability, including decreased number of teeth, reduced occlusal support in the molar region, decreased biting force, periodontal disease [10]. Reduced chewing ability leads to the avoidance of foods that are difficult to chew, which narrows the range of food choices and decreases dietary diversity [11]. Accordingly, there is a decreased intake of vegetables, fish, and meat, which leads to a deficiency of nutrients such as protein, sodium, calcium, vitamins, dietary fiber, folic acid, carotenoids, niacin, pantothenic acid, and iron [12,13]. These changes in nutrient intake increase the risk of malnutrition and frailty [14], as well as lifestyle-related diseases such as metabolic syndrome [15]. Furthermore, malnutrition impairs oral function through decreased muscle mass and strength, including masticatory and tongue muscles; weakening of the oral mucosal barrier function; altered taste and smell; and decreased salivary gland function. These effects accelerate the “frailty cycle” and increase the risk of requiring long-term care [16,17,18,19].

There have been several studies conducted at university hospitals to determine whether dental intervention improves nutritional status or whether nutritional guidance improves oral function. Salazar et al. reported that placement of new complete dentures improved masticatory performance and food intake, with both parameters being correlated [20]. Contrastingly, Suzuki et al. reported that providing complete dentures and simple nutritional guidance was more effective in increasing nutrient intake and improving masticatory function [21]. Hori et al. found that combining oral function training with nutritional guidance for 3 months improved both oral function and nutritional status in patients with oral hypofunction [22]. However, these reports did not include interventions that consisted solely of nutritional guidance, and reports on nutritional guidance in general dental clinics are even more limited [23].

General dental clinics serve as places where patients can receive regular checkups and oral care as well as address their lifestyle and dietary habits. Introducing nutritional guidance in collaboration with registered dietitians can provide dental care with a new role, which may effectively promote the prevention of oral functional decline. Accordingly, we aimed to examine the effects of simple nutritional guidance on oral function and body composition in patients at a general dental clinic where a registered dietitian was employed. Our null hypothesis was that 3 months of nutritional guidance would have no effect on improving oral function or body composition of patients who visit a single dental clinic for regular maintenance.

## 2. Materials and Methods

### 2.1. Participants

Between April 2024 and October 2025, we included patients aged ≥50 years who visited a dental clinic in Higashi-Osaka City, Osaka Prefecture for maintenance treatment. Individuals who provided informed consent to receive nutritional guidance were designated as the “guidance group (G group).” Patients who refused nutritional guidance but made regular clinic visits for maintenance at 3-month intervals were designated as the “no-guidance group (nG group).” After approval from the dental clinic, the study was announced via a notice posted at the clinic. Because this intervention was unprecedented at the time we began the study, the sample size calculations based on assumed effect sizes was not performed and instead the maximum number of participants possible were included. We excluded individuals with systemic diseases/disorders and bad habits that could seriously affect oral function, judged as lacking the capacity to provide consent owing to conditions such as dementia and those with a cardiac pacemaker.

### 2.2. Investigation Items

#### 2.2.1. General and Dental Items

For both groups, the following items were investigated at baseline: medical history, current medical condition, number of functional teeth, and presence and fit of dentures.

#### 2.2.2. Oral Function Assessment

For both groups, the following items from the diagnostic criteria of oral hypofunction were assessed at baseline and after 3 months. Oral function assessment was performed by a dentist (KM) under the guidance of an expert (TO), but the assessor was not blinded to whether or not the participants had received nutritional guidance.

##### Oral Moisture Level

The oral mucosal moisture level was measured at the center of the dorsal surface of the tongue, ≈10 mm from the tongue tip, using an oral moisture meter (Mucus, Life, Saitama, Japan).

##### Masticatory Performance

After chewing a gummy jelly (Gummy Jelly for masticatory performance assessment, UHA Mikakuto, Osaka, Japan) 30 times, the degree of comminution was evaluated through comparison with visual materials. For denture wearers, measurements were obtained with the dentures in place.

##### Tongue and Lip Motor Function

The number of pronunciations of each syllable /pa/, /ta/, and /ka/ in 5 s was measured using a tongue and lip motor function measuring device (Kenko-kun Handy; Takei Kiki Kogyo, Niigata, Japan). For denture wearers, measurements were obtained with the dentures in place.

##### Maximum Tongue Pressure

The maximum voluntary tongue pressure was measured by having the participant voluntarily press the tongue pressure probe connected to a tongue pressure measuring device (JMS Tongue Pressure Measuring Device; JMS, Hiroshima, Japan) between the tongue and palate with maximum force for several seconds. For denture wearers, measurements were obtained with the dentures in place.

##### Swallowing Function

The Repetitive Saliva Swallowing Test (RSST) was used to screen for the risk of aspiration. With the second and third fingers palpating the hyoid bone and thyroid cartilage, respectively, the participants were instructed to perform dry swallowing and the number of swallows within 30 s was measured.

#### 2.2.3. Body Composition Assessment

In the G group, body composition was assessed at baseline as well as at 1, 2, and 3 months using a body composition analyzer (ITO-InBody370, Ito Chotampa, Saitama, Japan). In the nG group, body composition was assessed at baseline and at 3 months. The body composition parameters included weight, body mass index (BMI), body fat percentage and mass, skeletal muscle mass, body water content, protein mass, mineral content, and basal metabolic rate.

#### 2.2.4. Questionnaire Survey on Changes in Dietary Habits

A questionnaire survey on changes in dietary habits was administered at the end of the intervention period. The participants were asked five questions regarding the type of food consumed, frequency of eating meat and fish, frequency of eating vegetables and fruits, frequency of eating chewy foods, and frequency of eating soft foods.

The responses were obtained using five options:

① Significantly increased

② Slightly increased

③ No change

④ Slightly decreased

⑤ Significantly decrease

### 2.3. Nutritional Guidance

Participants in the G group were asked to complete the Dietary Habits Questionnaire (meal frequency and times, food allergies, likes and dislikes, use of nutritional supplements, exercise habits, frequency, and amount of major food intake) and create a 3-day food diary at the baseline assessment. After 1 week, during their follow-up visit, after dental treatment, and while still in the dental chair, one registered dietitian with 4 years of experience who worked full-time at the dental clinic provided 5–10 min of nutritional guidance based on lifestyle habits, dietary content, oral function measurements, and body composition assessment results. This nutritional guidance was provided three times (at baseline as well as after 1 and 2 months), with a focus on the following three points:

1. To encourage the inclusion of one protein-containing food item in each of the three meals.
2. To encourage conscious nutritional replenishment after engaging in exercise habits such as walking or sports.
3. To encourage a balanced diet using the Dietary Variety Score table [24].

### 2.4. Statistical Analysis

A repeated-measures two-way analysis of variance (two-way ANOVA, time [baseline/after 3 months] × group [G-group/nG-group]) followed by the Bonferroni correction was performed to analyze the intervention effect on oral function and body composition. Between-group comparison of the change in dietary habit was performed using Fisher’s exact test. Statistical analyses were performed using R (version 4.5.1; R Foundation for Statistical Computing, Vienna, Austria). Statistical significance was set at *p* < 0.05.

## 3. Results

**Figure 1:**
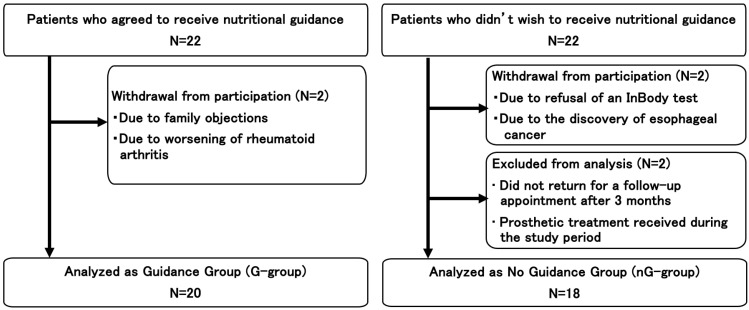
Flowchart of the enrollment of the participants.

During the study period, we invited 44 individuals (22 in each group). Subsequently, two participants in each group withdrew from the study, while two in the nG group were excluded. Therefore, the final analysis included 20 and 18 participants in the G and NG groups, respectively (Figure 1).

At baseline, the age and proportion of women in the nG group were higher than those in the G group, but no significant differences were observed. There were no significant between-group differences in terms of oral condition, oral function, or body composition (Table 1).

Three months after baseline, oral function showed an interaction between time and intervention (nutritional guidance) in masticatory performance (F(1, 36) = 13.03, *p* < 0.001), maximum tongue pressure (F(1, 36) = 6.58, *p* = 0.015), and RSST score (F(1, 36) = 8.44, *p* = 0.006) (Table 2). Additionally, a main effect of time was observed in masticatory performance and RSST score. Post hoc tests using Bonferroni correction showed that only in the G group, masticatory performance (*p* < 0.001), maximum tongue pressure (*p* = 0.005), and RSST score (*p* < 0.001) increased significantly after 3 months compared to baseline. On the other hand, no main effects or interactions between time and intervention were observed for body composition (Table 3).

The results of the questionnaire regarding eating habits were compared between the groups by dividing them into two categories: Increased (markedly/slightly) and others, taking into account the distribution of responses. In the G group, more than half of the participants reported an increase in the frequency of eating meat, fish, vegetables, and fruits; further, 45% reported an increase in the variety of foods consumed (Table 4). Additionally, 25% reported an increase in the frequency of eating foods with firm texture; however, no participant reported an increase in the frequency of eating soft foods. These changes in dietary habits were not observed in the nG group, with a significant between-group difference in all the subjective parameters except the frequency of eating soft foods (*p* < 0.05).

## 4. Discussion

To our knowledge, the effectiveness of nutritional guidance interventions in improving oral function in older patients remains unclear. Recently, Hori et al. reported that combining nutritional guidance and functional training was effective in improving nutritional intake status and reducing the number of symptoms of oral frailty in older patients diagnosed with oral frailty [22]. Sumino et al. found that nutritional guidance and functional training programs were effective in improving food intake diversity, tongue and lip movement, and swallowing function among older patients in general dental clinics [23]. The present study targeted patients receiving regular maintenance after treatment in a general dental clinic who received simple nutritional guidance once a month, which improved some test items relating masticatory and swallowing function after 3 months. Therefore, our null hypothesis is partially rejected.

Older individuals experience a decline in muscle protein synthesis capacity with age and exhibit anabolic resistance, which impedes improvements in muscle mass and function [25]. Furthermore, combining exercise with nutritional intervention has been shown to be more effective than nutritional guidance alone in improving muscle strength and mass [26,27,28]. Based on these findings, we provided practical guidance such as adding one protein-rich food item to each meal and encouraging nutritional supplementation after exercise. This guidance yielded an increase in the variety of consumed foods including chewy foods, which may have led to an increase in the chewing frequency and duration, suggesting that a functional training effect on the masticatory muscles was achieved [29].

Two-way ANOVA and post hoc test suggested the possibility that nutritional guidance improved oral function test scores related to masticatory and swallowing functions. Masticatory performance is influenced by tongue muscle function [30] as well as the number of teeth, occlusal status, occlusal force and periodontal disease. Accordingly, the observed improvement in maximum tongue pressure may have contributed to the improvement in the masticatory performance score. Given that the tongue is a muscular tissue, individuals with progressing sarcopenia might show a decrease in the maximum tongue pressure [31]. Resistance training is effective in improving tongue pressure [32]; however, the necessity for adequate protein intake has also been highlighted [33]. The RSST was originally developed to screen for the aspiration risk in stroke survivors [34]. In our study, the increase in the RSST score may reflect improved activity of swallowing-related muscle groups of the oral cavity and pharynx rather than changes in the cranial nerve mechanisms. These changes are merely speculative and do not prove a causal relationship between our intervention (nutritional guidance) and the improvement of oral function. Furthermore, the lack of a main effect of group in a two-way ANOVA indicates the weakness of the evidence.

We observed no postintervention improvements in the amount of oral moisture, which reflects salivary secretion function, or in the number of repetitions of oral diadochokinesis, which reflects the dexterity of tongue and lip movements. Although significant improvements in masticatory performance due to prosthetic treatment improve salivary secretion function [35], our observed changes in masticatory performance may have not been sufficiently substantial. Furthermore, Sumino et al. [23] reported that combined nutritional guidance with functional training improved both oral diadochokinesis and RSST, which suggests that these items are less likely to show improvement with nutritional guidance alone.

Several factors may explain the lack of significant postintervention changes in body composition parameters, including BMI, body fat percentage, skeletal muscle mass, and protein mass. First, we included patients who could independently visit the dental clinic, which excluded those with underlying diseases requiring dietary restrictions. Therefore, there were no significant differences in the body composition at baseline. Consequently, changes in dietary habits may have been insufficient to yield changes in body composition. Second, the nutritional guidance focused on dietary balance and strategies for protein intake; however, it did not involve strict guidance and management with quantitative intake targets. Therefore, participants may have not reached the recommended intake of 20–30 g of high-quality protein per meal, which is recommended to maximize muscle protein synthesis in individuals [36,37,38]. Furthermore, although the nutritional guidance recommended nutrient intake after exercise, it did not specify the precise amount of exercise required.

In this study, nutritional guidance was provided for 3 months to minimize the burden on participants. However, Sumino et al. observed no changes in body composition even after 6 months of oral function training and nutritional guidance [23]. Eglseer et al. reported that changes in muscle mass and body fat percentage were more likely to be achieved with continuous intervention for ≥6 months [27]. Therefore, the lack of change in body composition may be attributed to a combination of factors, including metabolic characteristics specific to older individuals, insufficient intensity of nutritional guidance, short intervention duration, and insufficient integration of exercise.

This study has several limitations. First, since this intervention method has not been previously used to address the outcome, it was difficult to predict the sample size. Second, although we included the maximum number of patients who consented to participate in the study, the resulting sample size was small and complete parity in age and gender ratios was not achieved. This impedes the generalizability of the results, and this study should be considered as a preliminary study to estimate effect sizes in future research. Furthermore, given the sex differences in physical and lifestyle factors, a larger sample size that allows for sex-stratified analyses is warranted. Third, we did not quantitatively evaluate changes in dietary habits as a result of nutritional guidance using monthly food records, which weakens the evidence regarding behavioral change. This is due to the variability in the completion rate of food records, which averaged 54% for every meal over the three-month period.

This study was conducted as a pilot study to examine the effects of simple nutritional guidance on oral function and body composition at a general dental clinic in Japan, and has various limitations. The results of this study were conducted on community-dwelling, independently living people aged 50 years or older, and may not apply to all older people. Future studies involving a larger, randomly assigned population are needed, with the intervention protocol (content and duration) reconsidered.

## 5. Conclusions

Although there are various limitations, a simple three-month nutritional guidance program for patients aged 50 years or older undergoing maintenance treatment at a general dental clinic in Japan has shown potential for maintaining and improving oral function.

## Figures and Tables

**Table 1:** Basic characteristics, oral function, and body composition in each group.

	Total	G Group	nG Group	*p* Value
Participants, n	38	20	18	
Age *	76.0 (69.0–78.8)	69.0 (62.8–78.0)	77.0 (75.3–78.8)	0.069
Male, n (%)	14 (36.8)	6 (30.0)	8 (44.4)	0.357
Denture wearers, n (%)	18 (47.4)	7 (35.0)	11 (61.1)	0.107
Number of functional teeth *	28.0 (28.0–28.0)	28.0 (27.0–28.0)	28.0 (28.0–29.0)	0.234
Number of remaining teeth *	24.0 (18.3–28.0)	25.0 (16.5–28.0)	23.0 (22.3–27.0)	0.565
Oral function *				
Masticatory performance	6.0 (3.3–7.0)	6.0 (3.8–7.0)	5.5 (3.5–7.0)	0.965
Phonation of /pa/, times/s	6.3 (5.8–6.8)	6.5 (6.0–6.9)	5.8 (5.6–6.7)	0.109
Phonation of /ta/, times/s	6.1 (5.6–6.6)	6.2 (5.8–7.0)	5.9 (5.6–6.4)	0.123
Phonation of /ka/, times/s	5.8 (5.4–6.4)	6.0 (5.6–6.4)	5.6 (5.0–6.2)	0.091
Tongue pressure, kPa	29.9 (26.1–34.4)	31.5 (27.3–36.5)	28.3 (24.3–32.9)	0.169
RSST, n/30 s	3.0 (3.0–3.8)	3.0 (2.8–4.0)	3.0 (3.0–3.0)	0.447
Oral moisture content	30.9 (29.1–32.3)	30.5 (29.3–32.6)	31.0 (28.5–32.0)	0.372
Body composition *				
Body weight, kg	53.7 (47.4–61.0)	53.3 (48.2–59.8)	54.6 (46.7–61.0)	0.803
BMI, kg/m^2^	21.6 (20.5–23.3)	21.5 (20.9–23.7)	22.1 (20.4–23.1)	0.861
Body fat percentage, %	28.9 (24.2–33.0)	29.8 (26.0–33.3)	26.1 (23.6–32.7)	0.306
Skeletal muscle mass, kg	20.5 (17.6–23.2)	20.5 (18.5–21.5)	21.5 (17.3–24.7)	0.474
Fat mass, kg	14.6 (13.1–18.8)	14.7 (14.2–19)	14.4 (11.6–17.8)	0.430
Fat-free mass, kg	38.3 (33.5–42.9)	38.3 (34.3–40.0)	40.0 (33.3–45.5)	0.474
Total body water, kg	28.2 (24.7–31.6)	28.2 (25.2–29.3)	29.5 (24.5–33.4)	0.474
Protein, kg	7.4 (6.5–8.4)	7.4 (6.8–7.8)	7.8 (6.4–8.9)	0.483
Minerals, kg	2.7 (2.4–3.0)	2.6 (2.4–2.9)	2.7 (2.4–3.1)	0.396
Basal metabolic rate, kcal	1197.0 (1094.3–1297.5)	1197.0 (1110.8–1234.8)	1232.5 (1089.3–1351.5)	0.474

* Median (IQR). Mann–Whitney U test.

**Table 2:** Change in the oral function between baseline and after 3 months in each group.

	G Group		nG Group		Main Effect		Interaction
					Time	Group	
	Baseline	3 Months	Baseline	3 Months	*p* Value	*p* Value	*p* Value
Masticatory performance	5.3 ± 2.4	6.5 ± 2.4	5.4 ± 2.4	5.3 ± 2.0	0.009	0.489	*p* < 0.001
Phonation /pa/, times/s	6.4 ± 0.8	6.5 ± 0.5	5.9 ± 1.0	6.1 ± 0.8	0.296	0.061	0.667
Phonation /ta/, times/s	6.3 ± 0.8	6.4 ± 0.6	5.8 ± 0.7	5.8 ± 0.8	0.226	0.024	0.491
Phonation /ka/, times/s	6.0 ± 0.6	6.2 ± 0.5	5.5 ± 0.9	5.6 ± 0.8	0.170	0.009	0.621
Tongue pressure, kPa	31.4 ± 8.6	33.9 ± 7.5	28.4 ± 8.2	27.8 ± 8.7	0.129	0.093	0.015
RSST, n/30 s	3.3 ± 1.0	4.1 ± 0.9	2.9 ± 0.5	3.0 ± 0.8	0.003	0.004	0.006
Oral moisture content	31.0 ± 2.0	30.0 ± 1.3	30.4 ± 1.9	30.4 ± 1.5	0.159	0.807	0.158

Mean ± standard deviation. Two-way ANOVA.

**Table 3:** Change in the body composition between baseline and after 3 months in each group.

	G Group		nG Group		Main Effect		Interaction
					Time	Group	
	Baseline	3 Months	Baseline	3 Months	*p* Value	*p* Value	*p* Value
Body weight, kg	55.1 ± 11.9	55.0 ± 11.9	56.2 ± 13.1	55.8 ± 13.0	0.202	0.811	0.514
BMI, kg/m^2^	22.2 ± 2.5	22.1 ± 2.7	22.0 ± 3.1	21.8 ± 2.8	0.065	0.816	0.941
Body fat percentage, %	29.6 ± 5.7	29.7 ± 6.0	26.9 ± 6.7	27.1 ± 6.2	0.638	0.183	0.896
Skeletal muscle mass, kg	20.7 ± 5.8	20.8 ± 5.7	22.1 ± 6.0	21.9 ± 6.4	0.730	0.526	0.469
Fat mass, kg	16.5 ± 4.6	16.3 ± 5.0	15.2 ± 5.4	15.1 ± 4.7	0.485	0.434	0.947
Fat-free mass, kg	38.6 ± 9.6	38.6 ± 9.4	41.0 ± 9.9	40.7 ± 10.7	0.587	0.491	0.460
Total body water, kg	28.4 ± 7.0	28.4 ± 6.9	30.2 ± 7.3	30.0 ± 7.9	0.672	0.479	0.500
Protein, kg	7.5 ± 1.9	7.5 ± 1.9	8.0 ± 2.0	7.9 ± 2.1	0.613	0.527	0.424
Minerals, kg	2.7 ± 0.6	2.7 ± 0.6	2.8 ± 0.6	2.8 ± 0.7	0.142	0.537	0.184
Basal metabolic rate, kcal	1203.1 ± 207.3	1204.2 ± 202.3	1254.8 ± 214.0	1248.9 ± 230.2	0.652	0.490	0.460

Mean ± standard deviation. Two-way ANOVA.

**Table 4:** Subjective assessment of the change in dietary habit.

		G Group	nG Group	*p* Value
Q1. Variety of foods	Increased (markedly/slightly)	9	0	0.001
	No change Decreased (slightly/markedly)	11 0	16 2	
Q2. Frequency of meat or fish consumption	Increased (markedly/slightly)	14	0	*p* < 0.001
	No change Decreased (slightly/markedly)	6 0	15 3	
Q3. Frequency of vegetable and fruit consumption	Increased (markedly/slightly)	13	0	*p* < 0.001
	No change Decreased (slightly/markedly)	7 0	18 0	
Q4. Frequency of chewy foods consumption	Increased (markedly/slightly)	5	0	0.048
	No change Decreased (slightly/markedly)	14 1	16 2	
Q5. Frequency of soft food consumption	Increased (markedly/slightly)	0	1	0.474
	No change Decreased (slightly/markedly)	20 0	17 0	

Fisher’s exact test.

## Data Availability

The data presented in this study are available upon request from the corresponding author. The data are not publicly available due to ethical restrictions.

## Abbreviations

The following abbreviations are used in this manuscript:

G group	Guidance group
nG group	No guidance group
RSST	Repetitive saliva swallowing test
